# Ameliorative Effect of *Beta vulgaris* Root Extract on Chlorpyrifos-Induced Oxidative Stress, Inflammation and Liver Injury in Rats

**DOI:** 10.3390/biom9070261

**Published:** 2019-07-07

**Authors:** Gadah Albasher, Rafa Almeer, Fatimah O. Al-Otibi, Noorah Al-Kubaisi, Ayman M. Mahmoud

**Affiliations:** 1 Department of Zoology, College of Science, King Saud University, Riyadh 11451, Saudi Arabia; 2 Department of Botany and Microbiology, College of Science, King Saud University, Riyadh 11451, Saudi Arabia; 3 Physiology Division, Zoology Department, Faculty of Science, Beni-Suef University, Beni Suef 62511, Egypt

**Keywords:** chlorpyrifos, red beetroot, oxidative stress, Nrf2, apoptosis

## Abstract

Exposure to organophosphorus insecticides causes several health problems to animals and humans. Red beetroot (RBR) is rich in antioxidant ingredients and possesses a promising hepatoprotective activity. This study evaluated the potential of RBR extract to prevent chlorpyrifos (CPF)-induced liver injury, with an emphasis on oxidative stress, inflammation and apoptosis. Rats received 10 mg/kg CPF and were treated with 300 mg/kg RBR extract for 28 days. CPF caused liver injury evidenced by elevated serum levels of serum alanine aminotransferase (ALT), aspartate aminotransferase (AST), alkaline phosphatase (ALP) and bilirubin, along with several histological alterations. Hepatic lipid peroxidation (LPO) and nitric oxide (NO) levels, as well as inducible nitric oxide synthase (iNOS) and pro-inflammatory cytokines were increased in CPF-intoxicated rats. RBR prevented CPF-induced histological alterations, and ameliorated liver function, LPO, NO, iNOS and pro-inflammatory cytokines. RBR boosted glutathione and antioxidant enzymes, and increased Nrf2 expression. In addition, RBR diminished Bax and caspase-3, and increased Bcl-2 expression. In conclusion, RBR prevented CPF-induced liver injury via attenuation of oxidative stress, inflammation and apoptosis. RBR enhanced antioxidant defenses, suggesting that it could be used as a potential therapeutic intervention to minimize CPF hepatotoxicity.

## 1. Introduction

Organophosphorus (OP) pesticides are a heterogeneous class of agricultural chemicals which are used to improve crop yields and agricultural productivity. Their application has increased annually throughout the world and in developing countries in particular [1]. The excessive use of OP pesticides was associated with high morbidity and mortality rates in farmers due to disorders resulting from acute and chronic exposure [2]. Ingestion, dermal absorption and inhalation are the main routes of exposure to OP pesticides [3]. Chlorpyrifos (CPF; [*O*,*O*-diethyl-o-(3,5,6-trichloro-2-pyridyl) phosphorothionate]) is one of the most currently used OPs. It is used to control household pests, flies and mosquitoes [4]. CPF is metabolized in hepatocytes by cytochrome P450 2B6 and converted to CPF oxon (oxygen analogue), which represents the major toxic CPF metabolite [5]. CPF acts as an acetylcholinesterase inhibitor [6]. However, several adverse effects have been strongly associated with its acute or chronic exposure, including reproductive dysfunction [7], developmental disturbances [8], neurotoxicity [9], cardiotoxicity [10], hematoxicity [11], nephrotoxicity and hepatotoxicity [12]. Although the mechanism underlying CPF toxicity is not fully understood, several reports have pointed to the implication of oxidative stress, inflammation and cell death [6,13]. Therefore, antioxidants have been suggested as potential therapeutic agents against CPF-induced oxidative reactions and subsequent side effects via quenching reactive oxygen species (ROS) [13]

*Beta vulgaris* or red beetroot (RBR) belongs to the Chenopodiaceae family and is widely grown in Europe, South America and Africa. It is used as a valuable vegetable, source of dyes and for medicinal proposes due to its high antioxidant property [14,15]. Numerous phytoconstituents have been identified in *B. vulgaris*, such as phenolic acids (ferulic, gallic, chlorogenic, vanillic, caffeic and syringic) and flavonoids (quercetin, myricetin, kaempferol and rutin) [16,17]. All parts of beetroot were found to have pharmacological activities, including antioxidant [16], anti-inflammatory, antitumor [18], blood pressure-lowering [19], neuroprotective [20] and immunomodulatory [21]. Hence, the current study was designed to assess whether RBR can prevent CPF hepatotoxicity by attenuating oxidative stress, inflammation and tissue injury.

## 2. Materials and Methods

### 2.1. Preparation of RBR Extract

Fresh RBR was collected from a local market in Riyadh, KSA in November 2018. Identification and authentication of the plant was carried out by a taxonomist. The roots were cleaned under tap water and ground using an electrical blender. The methanolic extract was prepared by macerating the obtained juice and particles three times in methanol (70%) for 48 h at a ratio of 1:10 (w/v) at 4 °C. The extract was filtered and the solvent was removed by vacuum evaporation followed by lyophilization. The RBR was maintained at −20 °C until further analysis.

### 2.2. Determination of Total Phenolics, Flavonoids and Radical Scavenging Activity of RBR

Total phenolics and flavonoids were determined using Folin Ciocalteu [22] and aluminum trichloride methods [23], respectively. The scavenging efficacy of RBR towards 2, 2-Diphenyl-1-picrylhydrazyl radicals (DPPH^•^) was tested in vitro as previously described [24], using DPPH supplied by Sigma (St. Louis, MO, USA).

### 2.3. Experimental Animals and Treatments

**Figure 1:**
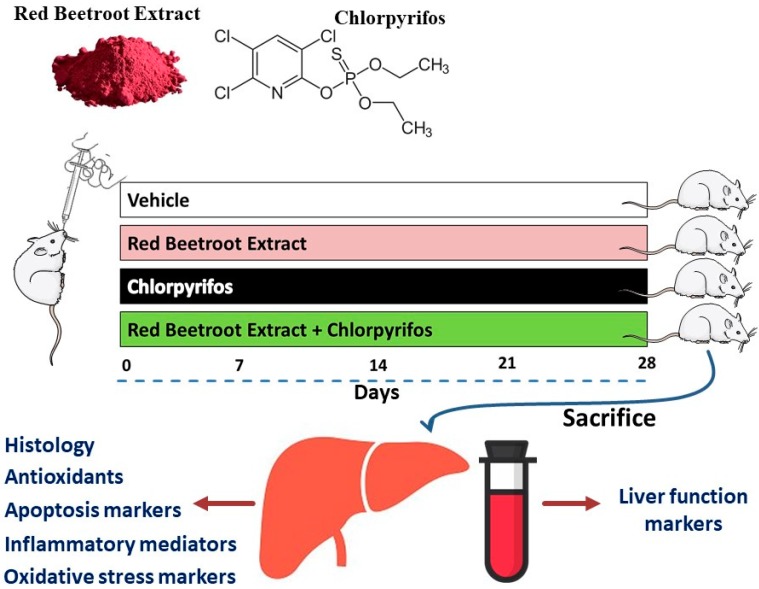
A schematic diagram showing the experimental design.

Twenty-eight adult Wistar rats (11-weeks-old; 140–160 g), obtained from the college of Pharmacy, King Saud University, were housed under standard conditions and supplied chow diet and water ad libitum. All experiments were approved by the Ethics Committee of King Saud University (Ethical approval no. H-01-R-059). The rats were randomly allocated into four groups (N = 7) as follows (Figure 1):

Group 1 (Control): Received distilled water 1 h before corn oil.

Group 2 (RBR): Received 300 mg/kg RBR [20] dissolved in distilled water.

Group 3 (CPF): Received 10 mg/kg CPF [25] dissolved in corn oil.

Group 4 (RBR + CPF): Received 300 mg/kg RBR [20] 1 h before the administration of 10 mg/kg CPF.

CPF (Pestban 48% EC) was supplied by Agro Chem (Alexandria, Egypt). Both RBR and CPF were administered *via* oral gavage daily for 28 days. Rats from all the groups were sacrificed under anesthesia, and blood was collected for serum preparation, and liver samples were removed immediately, weighed, and cut into pieces. The first piece was homogenized for biochemical investigations, the second piece was kept for RNA extraction, whereas the third piece was kept in neutral-buffered formalin (NBF) for histological studies.

### 2.4. Preparation of Tissue Homogenate and Assay of Protein Content

Liver samples were homogenized (10% *w*/*v*) in 50 mM Tris-HCl (pH 7.4) and centrifuged at 5000× *g* for 10 min at 4 °C. The obtained supernatant was stored at −80 °C until further processing in biochemical experiments. The protein content was assessed following the Lowry method [26] using bovine serum albumin (BSA) as a standard.

### 2.5. Biochemical Parameters

#### 2.5.1. Determination of Liver Function Markers

Serum alanine aminotransferase (ALT) [27], aspartate aminotransferase (AST) [27], alkaline phosphatase (ALP) [28] and total bilirubin [29] were assayed using commercially available assay kits (Biosystems, Spain).

#### 2.5.2. Determination of Oxidative Stress Biomarkers and Antioxidants

Thiobarbituric acid reactive substances (TBARS) [30], nitric oxide (NO) [31] and glutathione (GSH) [32] were assayed in the homogenate samples. Superoxide dismutase (SOD) [33], catalase (CAT) [34], glutathione peroxidase (GPx) [35] and glutathione reductase (GR) [36] activities were estimated according to the previously described methods.

#### 2.5.3. Determination of Pro-Inflammatory Cytokines

Tumor necrosis factor (TNF)-α and interleukin (IL)-1β were assayed in the liver homogenates using ELISA kits (ThermoFisher Scientific, Waltham, MA, USA; Cat. No. ERIL1B and R&D Systems, Minneapolis, MN, USA; Cat. No. RTA00, respectively), following the manufacturers’ instructions.

#### 2.5.4. Determination of Apoptosis Markers

Cytochrome *c*, Bax and Bcl-2 were determined using ELISA kits obtained from Cusabio (Wuhan, China; Cat. No. CSB-EL006328RA), BioVision, Inc. (Milpitas, CA, USA; Cat. No. E4513) and Cusabio (Wuhan, China; Cat. No. CSB-E08854r), respectively. Caspase-3 activity was measured using a colorimetric kit (Sigma-Aldrich, St. Louis, MO, USA; Cat. No. CASP3C-1KT).

### 2.6. Gene Expression Analysis

Total RNA was isolated from the frozen samples using TRIzol^®^ reagent (Invitrogen, Waltham, MA, USA). The isolated RNA was quantified and immediately reverse transcribed to cDNA which was amplified using SYBR^®^ Green Master Mix and the primers listed in Table 1 [37,38]. The PCR amplification included initial denaturation for 10 min at 95 °C and 40 cycles (94 °C for 10 s, annealing at 60 °C for 30 s, and extension at 72 °C for 20 s). The data were analyzed using the 2^−ΔΔ*C*t^ method [39] and normalized to GAPDH.

### 2.7. Histology and Immunohistochemistry

Liver samples were fixed 10% NBF for 24 h and processed for paraffin embedding. After cutting, the 5 μm sections were stained with hematoxylin and eosin (H&E) and examined using a Nikon microscope (Eclipse E200-LED, Tokyo, Japan). Other sections were stained with anti-Bax as previously described [40,41]. Briefly, the tissue sections were blocked in 3% hydrogen peroxide (H_2_O_2_) and then probed with a rabbit polyclonal Bax antibody overnight at 4 °C. The sections were washed and probed with a biotinylated secondary antibody, followed by streptavidin/peroxidase conjugate for 30 min and then diaminobenzidine. The sections were counterstained with hematoxylin and examined using a Nikon microscope (Eclipse E200-LED, Tokyo, Japan).

### 2.8. Statistical Analysis

The obtained results were expressed as means ± standard deviation (SD). The statistical comparisons were made by one-way ANOVA followed by a Tukey’s test using Graphpad Prism 7 (La Jolla, CA, USA). A *p* value < 0.05 indicates a statistical significance.

## 3. Results

### 3.1. Total Phenolics, Flavonoids Content and DPPH Radical Scavenging Activity of RBR

**Figure 2:**
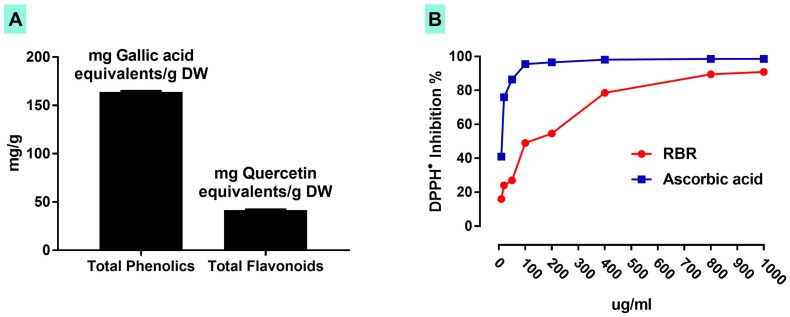
Total phenolics and flavonoids content (**A**) and DPPH• scavenging activity of red beetroot (RBR) extract (**B**). Data are the mean values of triplicate and expressed as mean ± SD.

Data represented in Figure 2A showed that the methanolic extract of RBR contains 164 ± 1.11 mg gallic acid equivalents/g dry weight (DW) total phenolics and 41.59 ± 0.89 mg quercetin equivalents/g DW flavonoids. The scavenging activity of RBR against DPPH^•^ was tested in vitro where it showed a concentration-dependent efficacy (Figure 2B).

### 3.2. RBR Prevents Liver Injury in CPF-Induced Rats

**Figure 3:**
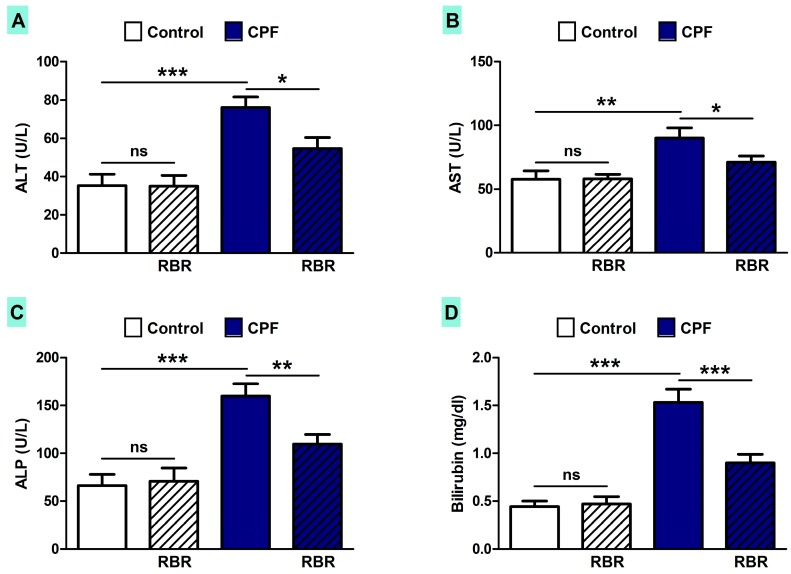
RBR prevents liver injury in chlorpyrifos (CPF)-induced rats. RBR ameliorates serum (**A**) ALT, (**B**) AST, (**C**) ALP and (**D**) bilirubin in CPF-intoxicated rats. Data are mean ± SD, (N = 7). * *p* < 0.05, ** *p* < 0.01 and *** *p* < 0.001. ns = non-significant.

The effect of RBR on liver function and histological structure was assessed in both control and CPF-induced rats. CPF triggered remarkable increase in serum ALT (*p* < 0.001; Figure 3A), AST (*P* < 0.01; Figure 3B), ALP (*p* < 0.001; Figure 3C) and bilirubin levels (*p* < 0.001; Figure 3D). RBR noticeably ameliorated liver function markers in CPF-induced rats, with no effect on these markers in the normal rats.

**Figure 4:**
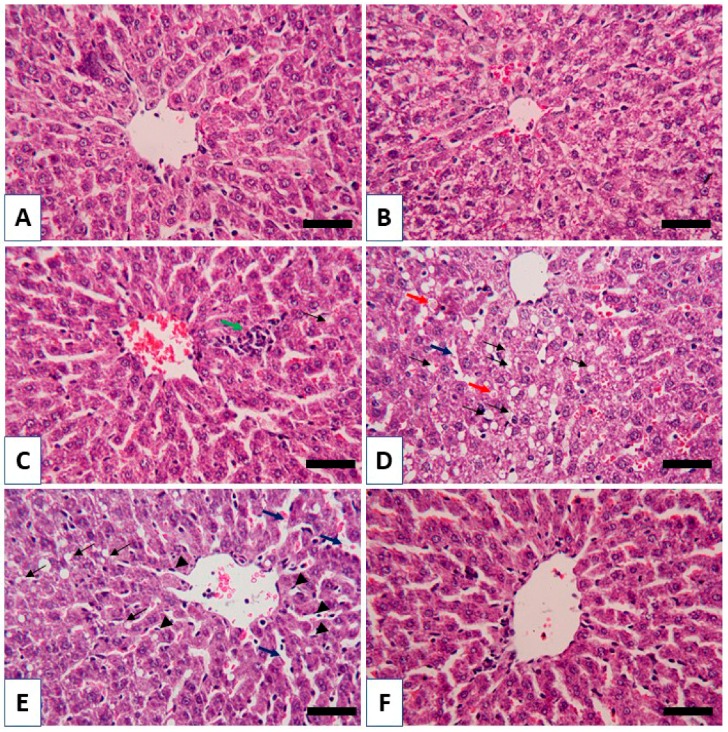
RBR inhibits histological alterations in CPF-intoxicated rats. Photomicrographs of sections in liver of (**A**) control, (**B**) RBR-supplemented groups showing normal hepatic lobules and hepatocytes, (**C**–**E**) CPF-intoxicated rats showing leukocyte infiltration (green arrow), vacuolations (black arrow), cytoplasmic vacuolations with eosinophilic substance (red arrow), dilated sinusoids (blue arrow) and Kupffer cells (arrow head) and (**F**) CPF-intoxicated rats treated with RBR showing no histological alterations. (X400, Scale bar 50 µm.).

The protective efficacy of RBR was supported by the histological findings. Both the control (Figure 4A) and RBR-supplemented rats (Figure 4B) showed normal histological architecture of hepatic lobules and hepatocytes. In contrast, CPF-induced rats showed several alterations, including leukocyte infiltration, dilated sinusoids, vacuolations and cytoplasmic vacuolations with eosinophilic substance (Figure 4C–E). RBR supplementation prevented CPF-induced histological alterations as represented in Figure 4F.

### 3.3. RBR Ameliorates Oxidative/Nitrative Stress and Improves Redox Homeostasis in CPF-Induced Rats

**Figure 5:**
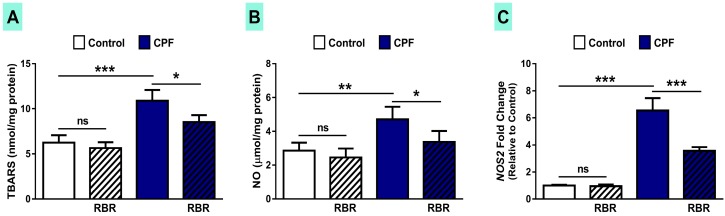
RBR prevents oxidative/nitrative stress and down-regulates iNOS in CPF-induced rats**.** RBR diminishes (**A**) TBARS, (**B**) NO and (**C**) iNOS mRNA expression in the liver of CPF-intoxicated rats. Data are mean ± SD, (N = 7). * *p* < 0.05, ** *p* < 0.01 and *** *p* < 0.001. ns = non-significant.

**Figure 6:**
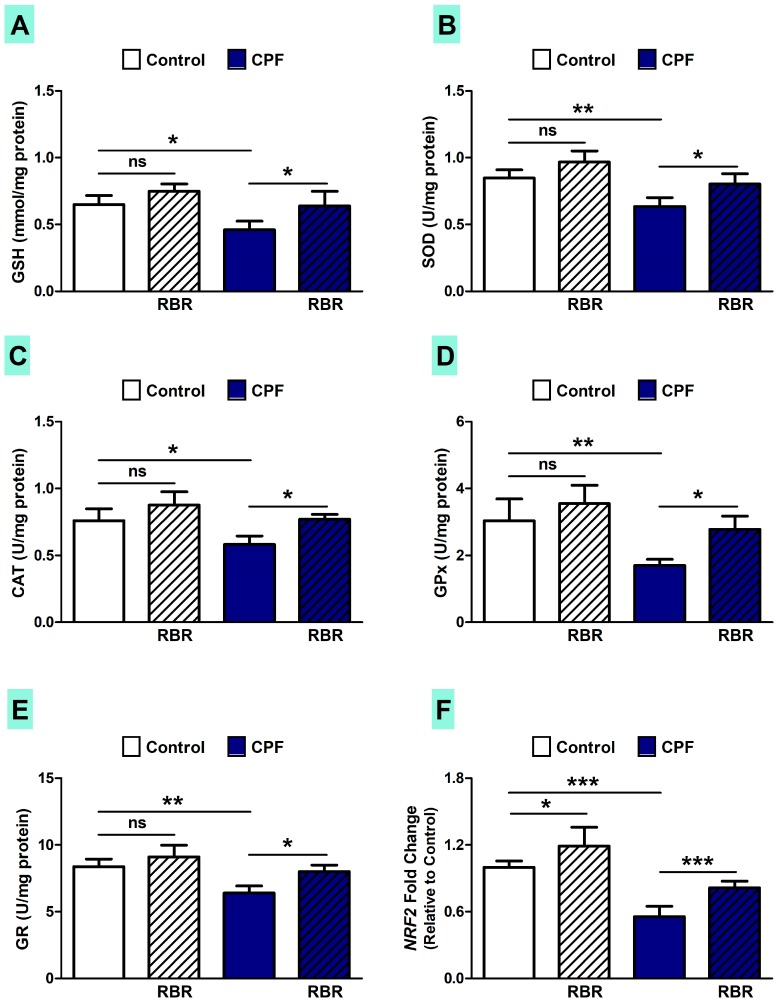
RBR enhances cellular antioxidants and up-regulates Nrf2 in control and CPF-induced rats. RBR increases (**A**) GSH content, and activity of (**B**) SOD, (**C**) CAT, (**D**) GPx and (**E**) GR in the liver of CPF-intoxicated rats. (**F**) RBR increased hepatic *Nrf2* expression. Data are mean ± SD, (N = 7). * *p* < 0.05, ** *p* < 0.01 and *** *p* < 0.001. ns = non-significant.

To assess redox homeostasis following CPF exposure and the ameliorative effect of RBR, we determined oxidative/nitrative stress markers (Figure 5) and antioxidant defenses (Figure 6). Rats exposed to CPF displayed a marked increase in TBARS (*p* < 0.001; Figure 5A), NO (*p* < 0.01; Figure 5B) and the expression of inducible nitric oxide synthase (iNOS; *p* < 0.001; Figure 5C). While its supplementation caused no effect in normal rats, RBR significantly diminished TBARS, NO and iNOS in CPF-induced rats.

The administration of CPF for 28 days resulted in declined hepatic GSH content (*p* < 0.05; Figure 6A) and the activity of SOD (*p* < 0.01; Figure 6B), CAT (*p* < 0.05; Figure 6C), GPx (*p* < 0.01; Figure 6D) and GR (*p* < 0.01; Figure 6E). RBR supplementation enhanced hepatic antioxidants in CPF-induced rats (*p* < 0.05), whereas it had no effect on the antioxidant defenses in normal rats. Given the central role of nuclear factor erythroid 2-related factor 2 (Nrf2) in promoting the expression of defensive enzymes [42], we thought that it might be involved in the ameliorative effect of RBR. CPF suppressed Nrf2 mRNA (*p* < 0.001), an effect that was reversed in RBR-supplemented rats (*p* < 0.001), as depicted in Figure 6F. Interestingly, RBR increased Nrf2 mRNA abundance in normal animals (*p* < 0.05).

### 3.4. RBR Attenuates Inflammation in CPF-Induced Rats

**Figure 7:**
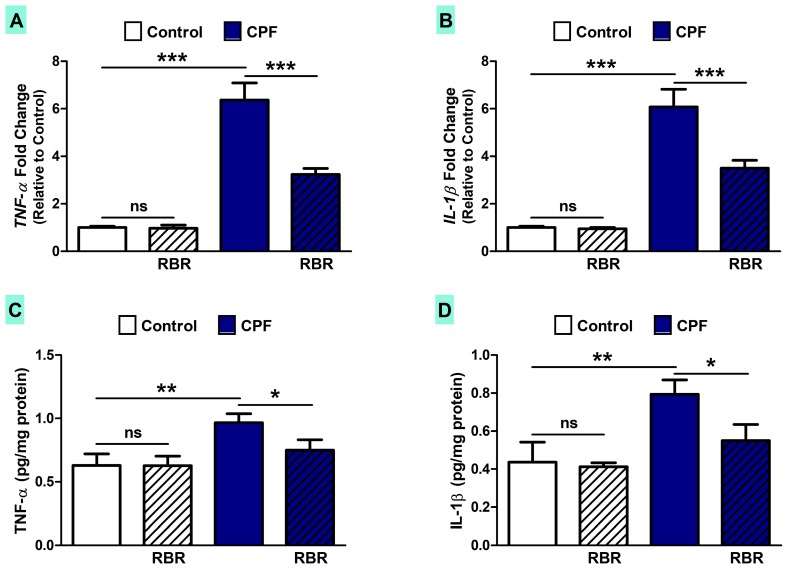
RBR attenuates inflammation in CPF-intoxicated rats. RBR decreases the gene (**A** and **B**) and protein expression (**C** and **D**) of TNF-α and IL-1β in the liver of CPF-intoxicated rats. Data are mean ± SD, (N = 7). * *p* < 0.05, ** *p* < 0.01 and *** *p* < 0.001. ns = non-significant.

TNF-α (Figure 7A) and IL-1β (Figure 7B) were up-regulated (*p* < 0.001) in the liver of CPF-intoxicated rats when compared with the control rats. These findings were confirmed by the ELISA assays where the liver of rats exposed to CPF showed an increase in TNF-α (*p* < 0.01; Figure 7C) and IL-1β (*p* < 0.01; Figure 7D). RBR supplementation markedly ameliorated TNF-α and IL-1β in CPF-induced rats, with no effect in normal rats.

### 3.5. RBR Mitigates Apoptosis in CPF-Induced Rats

**Figure 8:**
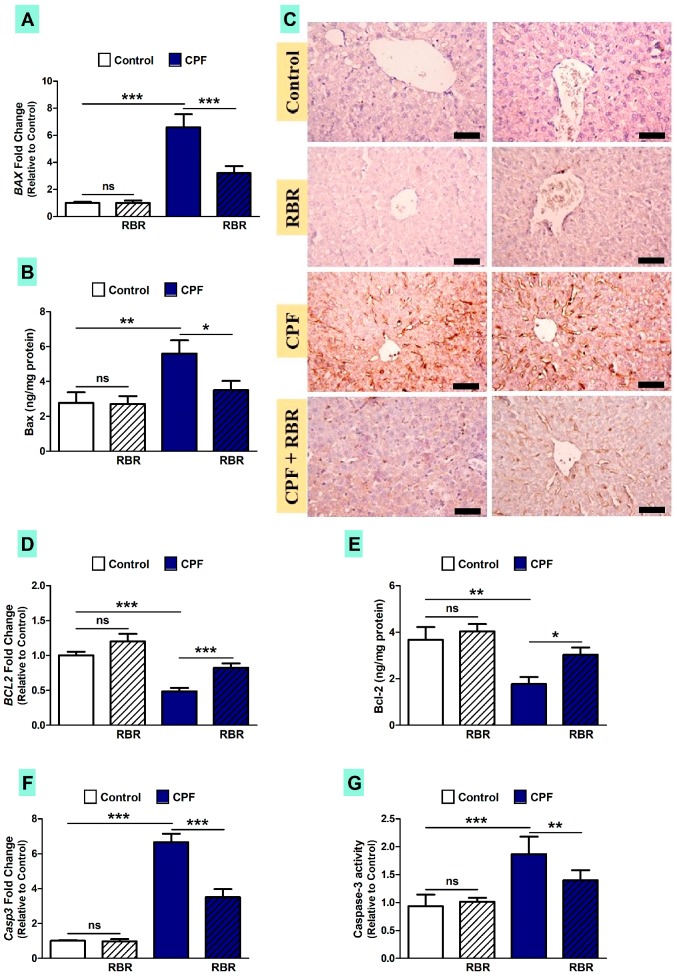
RBR mitigates apoptosis in CPF-induced rats. RBR decreases Bax mRNA (**A**) and protein (**B**) and caspase-3 (**F** and **G**). (**C**) Photomicrographs of the immunohistochemical staining of Bax showing increased Bax in the liver of CPF-intoxicated rats and the alleviative effect of RBR. (400×, Scale bar 50 µm). (**D** and **E**) RBR increased Bcl-2 in CPF-intoxicated rats. Data are mean ± SD, (N = 7). * *p* < 0.05, ** *p* < 0.01 and *** *p* < 0.001. ns = non-significant.

Owing to the positive correlation between inflammation, oxidative stress and apoptosis in animals exposed to hepatotoxins [43,44,45,46], we determined the expression of apoptosis markers in the liver of CPF-administered rats. CPF up-regulated Bax mRNA (*p* < 0.001; Figure 8A) and protein levels determined by both ELISA (*p* < 0.01; Figure 8B) and immunohistochemistry (Figure 8C), as well as caspase-3 (*p* < 0.001; Figure 8E; *p* < 0.001; Figure 8F). In contrast, CPF decreased Bcl-2 mRNA and protein levels (*p* < 0.001; Figure 8C; *p* < 0.01; Figure 8D). RBR significantly ameliorated Bax, Bcl-2 and caspase-3 levels in CPF-administered rats. Oral administration of RBR did not cause changes in the expression of apoptosis markers in normal rats (Figure 8).

## 4. Discussion

CPF has been classified among the most used OP pesticides for agricultural and household purposes and its application has been linked with severe adverse reactions in farm animals and humans. This investigation was conducted to study the potential protective efficacy of RBR on CPF hepatotoxicity. CPF-intoxication provoked an elevation in the liver function markers, which is a direct consequence of hepatocyte injury as described previously [47]. CPF hepatotoxicity was affirmed by the histological findings, including infiltration of leukocytes, vacuolations, degenerative changes, proliferation Kupffer cells and other manifestations. Hepatocyte degeneration and apoptosis, as well as inflammatory cell infiltration were established in the CPF-intoxicated rats, which could be explained primarily by the formation of ROS [48]. Interestingly, oral supplementation of RBR to CPF intoxicated rats ameliorated liver function and prevented all histological alterations, demonstrating its potent hepatoprotective efficacy. RBR has been recently reported to protect against carbon tetrachloride hepatotoxicity via restoration of histological and biochemical changes [49,50].

The hepatoprotective effect of RBR could be attributed to attenuation of oxidative damage and improvement of cellular antioxidants. Earlier studies have reported that exposure to OP pesticides, including CPF, disrupts the oxidant/antioxidant detoxification system which consequently causes peroxidation of the cell membrane lipids and reduces enzymatic and non-enzymatic antioxidants in vital organs of experimental animals [47,48]. Meanwhile, the consequences of OP pesticides were improved by exogenous antioxidant treatment. Our study revealed that CPF intoxication diminished SOD, CAT, GPx and GR, and triggered lipid peroxidation (LPO). Additionally, chronic exposure to CPF enhanced NO generation in the liver, an effect that was explained by the up-regulated iNOS. The expression of iNOS is controlled by transcriptional (nuclear factor-kappaB [NF-κB]) as well as post-transcriptional mechanisms [51]. Although NO is involved in regulating various physiological processes, iNOS-mediated generation of NO can result in the production of peroxynitrite which induces oxidative DNA damage and cell injury [52]. Moreover, CPF reduced GSH content and antioxidant enzymes in the liver of rats. The reduction in GSH level is due to its use to conjugate electrophilic metabolites of CPF as well as to counteract the overproduction of ROS and LPO [53]. Furthermore, excess generation of ROS inflicts damage to nearly all bio-macromolecules, such as DNA, lipids and proteins, leading to cytotoxicity. Excessive generation of ROS provoked by the exposure to OP pesticides may also interrupt genetic integrity and modify the biochemistry of metabolic pathways. The diminished activity of SOD in liver of CPF-intoxicated rats could be explained in terms of its excessive use in the dismutation of superoxide radicals to H_2_O_2_. CAT and GPx can then decompose H_2_O_2_ into oxygen and water, and GR promotes the NADPH-driven conversion of oxidized glutathione to the reduced form which is used as a substrate for GPx. Therefore, these antioxidant defenses confer protection against ROS-induced oxidative damage. Moreover, enhancement of this cellular defense system can provide an effective strategy to prevent CPF-induced oxidative stress and liver injury. Interestingly, RBR attenuated LPO, iNOS expression and NO production, and boosted GSH and antioxidant enzymes.

The antioxidant activity of RBR may be due to its high content of phenolics, flavonoids and the red colorant betanin. The radical-scavenging efficacy of betanin has been previously demonstrated. In diabetic rats, betanin attenuated renal fibrosis [54] and protein glycation [55] by preventing oxidative stress. The antioxidant activity of betanin has also been confirmed by in vitro cell culture experiments [56]. Betanin’s exceptional electron donating capacity has been reported as the main reason of its high antioxidant capacity [57]. In addition to its radical scavenging efficacy, betanin has induced the antioxidant defenses via activation of Nrf2 signaling [56]. These findings add support to our results where RBR up-regulated Nrf2 in normal and CPF-intoxicated rats. Nrf2 plays a crucial role in controlling endogenous antioxidants in response to oxidative stress. The protein conformational changes induced by ROS can dissociate Nrf2 from Kelch-like ECH-associated protein 1 (Keap1) followed by its translocation into the nucleus and binding to the antioxidant responsive element (ARE), promoting the expression of antioxidant and defensive genes [42]. Hence, Nrf2 expression regulates the adaptive response of cells toward a variety of oxidants and electrophiles. Although activated by ROS, Nrf2 has been reported to be diminished by excessive ROS [58,59,60]. Herein, RBR prevented CPF liver injury via its dual ability to scavenge ROS and activate Nrf2. The antioxidant efficacy of RBR has been confirmed through its ability to scavenge DPPH^•^ in vitro. It is noteworthy to mention that RBR contains other phenolic compounds, including epicatechin, caffeic acid and rutin, with well-acknowledged antioxidant activity [61,62].

Besides oxidative stress, CPF intoxication in rats resulted in increased TNF-α and IL-1β, which might be attributed to ROS-mediated activation of NF-κB. This notion is supported by a recent study demonstrated NF-κB activation in CPF/lipopolysaccharide-challenged neonate rats [63]. Additionally, exposure to CPF increased pro-inflammatory cytokines in brain, ovary and uterus tissue of rats [64]. Moreover, Jang et al. [65] reported that CPF-mediated mitochondrial oxidative stress can stimulate the inflammasome and subsequent innate immune response, suggesting that ROS scavengers might be used to repair damage caused by pesticides. In this regard, RBR and its active ingredients have attracted attention as powerful anti-inflammatory agents. RBR administration for 28 days to rats attenuated NF-κB DNA-binding activity [66], and betalain-rich oral capsules alleviated pain and inflammation in osteoarthritis patients [67]. Activation of Nrf2 can suppress NF-κB and mitigate inflammation [68], and is therefore involved in the ameliorative effect of RBR

Exposure to CPF increased Bax and caspase-3, and decreased Bcl-2 expression, demonstrating apoptosis. Previous studies have demonstrated up-regulated pro-apoptotic mediators in the liver of rats [13] and in common carp gills [69] following exposure to CPF. In addition, Chen et al. [70] showed the role of ROS and phosphorylated-AMP-activated-protein-kinase (p-AMPK) in CPF-induced testicular cell apoptosis. Oral supplementation of RBR regulated the expression of pro- and anti-apoptosis mediators. Accordingly, El Gamal et al. [66] have demonstrated up-regulation of anti-apoptotic proteins and down-regulated pro-apoptotic proteins in gentamicin-induced rats treated with RBR. The anti-apoptotic efficacy of RBR was explained via its radical-scavenging, antioxidant and anti-inflammatory activities.

## 5. Conclusions

**Figure 9:**
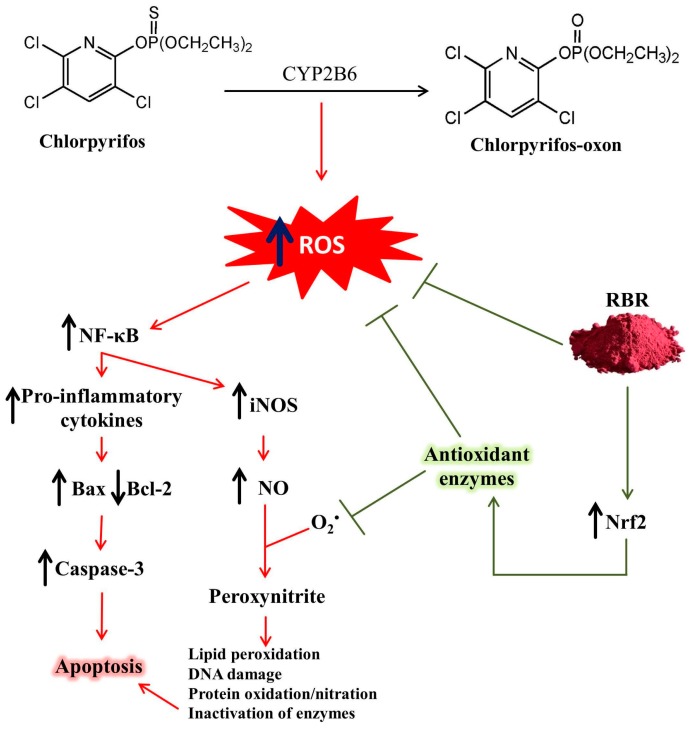
A proposed schematic diagram illustrating the protective mechanism of RBR against CPF hepatotoxicity.

Exposure to CPF elicits liver injury mediated via oxidative stress, inflammation and cell death. CPF provoked functional and histological alterations, lipid peroxidation, pro-inflammatory cytokines production and up-regulation of pro-apoptotic mediators. In addition, CPF diminished antioxidant defenses, Nrf2 and Bcl-2. RBR prevented CPF-mediated liver injury through its ability to enhance Nrf2 expression and cellular antioxidants, and attenuate LPO, inflammation and apoptosis (Figure 9). Therefore, RBR could be exploited as a potential therapeutic intervention to minimize CPF-induced hepatic injury, pending further studies to explore the exact mechanisms underlying its hepatoprotective activity.

## Figures and Tables

**Table 1:** Primers used for qRT-PCR.

Gene	Genbank Accession Number	Sequence (5′-3′)
*BAX*	NM_017059.2	F: GGGCCTTTTTGCTACAGGGT R: TTCTTGGTGGATGCGTCCTG
*BCL2*	NM_016993	F: ACTCTTCAGGGATGGGGTGA R: TGACATCTCCCTGTTGACGC
*NOS2*	NM_012611.3	F: GTTCCTCAGGCTTGGGTCTT R: TGGGGGAACACAGTAATGGC
*TNFα*	NM_012675.3	F: GGCTTTCGGAACTCACTGGA R: CCCGTAGGGCGATTACAGTC
*Il1β*	NM_031512.2	F: GACTTCACCATGGAACCCGT R: GGAGACTGCCCATTCTCGAC
*Nfe2l2*	NM_031789.2	F: TTGTAGATGACCATGAGTCGC R: ACTTCCAGGGGCACTGTCTA
*Casp3*	NM_012922.2	F: GAGCTTGGAACGCGAAGAAA R: TAACCGGGTGCGGTAGAGTA
*Gapdh*	NM_017008.4	F: AGTGCCAGCCTCGTCTCATA R: GATGGTGATGGGTTTCCCGT

*Gapdh*, glyceraldehyde-3-phosphate dehydrogenase; *Nfe2l2*, nuclear factor erythroid 2-related factor 2; *NOS2*, inducible nitric oxide synthase; *Il1β*, interleukin 1 beta; *Tnf*, tumor necrosis factor; *Bcl2*: B-cell lymphoma 2; *Bax*, Bcl-2-like protein 4; *Casp3*, caspase-3.
